# Human-in-the-loop assisted de novo molecular design

**DOI:** 10.1186/s13321-022-00667-8

**Published:** 2022-12-28

**Authors:** Iiris Sundin, Alexey Voronov, Haoping Xiao, Kostas Papadopoulos, Esben Jannik Bjerrum, Markus Heinonen, Atanas Patronov, Samuel Kaski, Ola Engkvist

**Affiliations:** 1 Department of Computer Science, Aalto University, Espoo, Finland; 2 Molecular AI, Discovery Sciences, R&D, AstraZeneca, Gothenburg, Sweden; 3 Department of Computer Science, University of Manchester, Manchester, UK; 4 Department of Computer Science and Engineering, Chalmers University of Technology, Gothenburg, Sweden; 5 Present Address: Odyssey Therapeutics, Cambridge, MA USA

**Keywords:** Interactive algorithms, De novo molecular design, Human-in-the-loop, AI-assisted design, Goal-oriented molecule generation, Expert knowledge elicitation, Reward elicitation

## Abstract

A de novo molecular design workflow can be used together with technologies such as reinforcement learning to navigate the chemical space. A bottleneck in the workflow that remains to be solved is how to integrate human feedback in the exploration of the chemical space to optimize molecules. A human drug designer still needs to design the goal, expressed as a scoring function for the molecules that captures the designer’s implicit knowledge about the optimization task. Little support for this task exists and, consequently, a chemist usually resorts to iteratively building the objective function of multi-parameter optimization (MPO) in de novo design. We propose a principled approach to use human-in-the-loop machine learning to help the chemist to adapt the MPO scoring function to better match their goal. An advantage is that the method can learn the scoring function directly from the user’s feedback while they browse the output of the molecule generator, instead of the current manual tuning of the scoring function with trial and error. The proposed method uses a probabilistic model that captures the user’s idea and uncertainty about the scoring function, and it uses active learning to interact with the user. We present two case studies for this: In the first use-case, the parameters of an MPO are learned, and in the second use-case a non-parametric component of the scoring function to capture human domain knowledge is developed. The results show the effectiveness of the methods in two simulated example cases with an oracle, achieving significant improvement in less than 200 feedback queries, for the goals of a high QED score and identifying potent molecules for the DRD2 receptor, respectively. We further demonstrate the performance gains with a medicinal chemist interacting with the system.

**Graphical Abstract:**

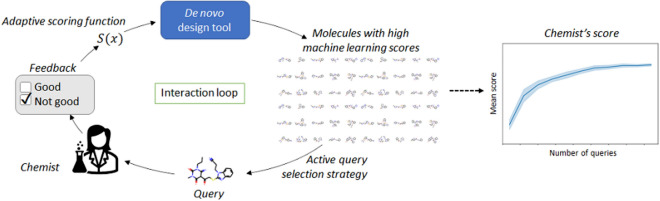

**Supplementary Information:**

The online version contains supplementary material available at 10.1186/s13321-022-00667-8.

## Introduction

The use of artificial intelligence and machine learning (AI/ML) in drug discovery has increased rapidly in recent years, providing AI-aided design tools for drug design projects [1–3]. The strengths of AI lie in finding patterns from vast amount of data from heterogeneous sources, at its best augmenting humans' abilities in challenging tasks such as molecular optimization. Advances in *de novo* molecular design tools enable automation of the design step in *in silico* design-make-test-analyze (DMTA) cycles of drug design [4, 5]. They transform the task of a chemist from designing a molecule to designing a scoring function that is used to evaluate the generated molecules, and which essentially expresses the chemist’s goal in a drug design project. Even though designing the scoring function may be an easier task for a human than coming up with new molecules, that is difficult, too, and AI/ML drug design tools to date do not provide aid for this task. In the current practice, a design tool generates a batch of molecules, which are filtered and evaluated by a chemist, who consequently manually tunes the scoring function and its parameters to yield better generated molecules. This iterative process is laborious and requires broad expertise. Furthermore, even after automatic post-processing filters, the number of generated molecules is in the hundreds or thousands, an order of magnitude higher than the number of molecules humans can feasibly evaluate.

We propose to assist this manual trial-and-error approach in designing the scoring function by interactive human-in-the-loop machine learning. *Human-in-the-loop learning (HITL)* is a branch of machine learning where human users can interact with a machine learning model during model training and usage, to integrate expert knowledge to the model and improve the model’s performance [6–8]. In molecular design, recent studies have found that medicinal chemist’s intuition can perform on par with machine learning methods e.g. in solubility prediction [9], but to the best of our knowledge human intuition has not been incorporated in a systematic way into *de novo* molecular design. The HITL approach introduced in this work provides a principled way for integrating human intuition into *de novo* molecular design.

Drug discovery is an inherently multi-objective problem where numerous pharmaceutically important objectives need to be satisfied, with the added complications that often objectives can be: i. Conflicting (for example in a project where increased solubility and increased metabolic stability are required–even though increasing solubility can cause reducing metabolic stability), ii. Challenging to quantify or measure experimentally (e.g. drug-likeness [10], synthetic accessibility objectives [11, 12]), and iii. The number of all potentially relevant objectives can be very large making the optimization landscape infeasible for most optimization algorithms; hence in practice a subset of objectives is usually selected and may need to be modified during the optimization process.

The concept of multiparameter optimization (MPO), is widely used in the context of medicinal chemistry [13–15]. For example, Wager et al. [15] used MPO for the central nervous system drug property space, by calculating scalar score as an empirical non-linear function of six fundamental physicochemical properties. Similar approaches have been widely applied by medicinal chemists in other therapeutic areas. Alternative approaches to address multi-objective optimization in drug design have been reported, where either the problem is transformed to a single objective by linear or non-linear weighting of the objectives, or a full or partial Pareto solution space is obtained; see a recent review by Nicolaou et al. [14]. Yasonik [16] recently suggested nondominated sorting and transfer learning to iteratively fine-tune a recurrent neural network, without a scoring function.

Typical scoring components in MPO include physical, chemical and predicted properties of a molecule, and desirability functions are used to define which values are preferred for each property. Once the scoring function is known, various machine learning methods can be used to explore the chemical space and generate novel molecular structures, including Monte Carlo tree search based on SMILES (simplified molecular-input line-entry system) strings [17], reinforcement learning [4], Generative Adversarial Networks (GANs) [5], genetic algorithms[18, 19], and Particle Swarm Optimization [20]. In other fields, previous work exists on interactively optimizing multiple objectives [21, 22], but these methods are limited to relatively low-dimensional design problems.

For designing the interaction with a chemist, an important question is which molecules should be presented (“queried”) to them for feedback and in which order. This type of problem is widely studied in active learning, automatic experimental design, and optimization. Active learning methods consider which new training instance to add to a supervised learning training set, to best improve the model’s accuracy [23]. In contrast, for optimization tasks a query must balance between exploration to learn about the problem, and exploitation to restrict the queries to potentially relevant ones. Simple exploration-exploitation problems can be formulated as so-called bandit problems (see e.g. [24]), and solved with methods that guarantee small cumulative regret, i.e. that minimize the loss from not querying the optimal items. Theoretical guarantees have been derived for linear [25], generalized linear [26, 27], and Gaussian process reward models [28], among others. A popular heuristic to solve the exploration-exploitation problem in more complex models is Thompson sampling, which chooses the action that maximizes the expected reward with respect to a randomly drawn belief [29, 30]. In case the humans are assumed to have knowledge about predictive features, previous HITL methods have shown that Bayesian sequential experimental design is effective in finding relevant features to a prediction task [7, 31].

Interactive multi-parameter optimization of molecules starts to raise interest, but to date few works exist. One example is grünifai [32], which optimizes molecules in a continuous vector space, starting from an input molecule, and allows a user to observe intermediate result molecules and give feedback (good/not good) to them. However, grünifai does not use an optimization strategy to select molecules that are shown to the user. In this work we compare different strategies to select molecules, and show their effect on the outcome of optimization, especially on the number of queries to the human needed to reach a goal. To our knowledge there is no proof of concept that interaction with a human chemist helps optimization, which is the key contribution in this work.

The way human feedback is incorporated in the MPO objective could be different as well. In this work we study two tasks. In Task 1, we use human feedback to infer the parameters of the desirability function of each component in an MPO function. In Task 2, we use human feedback to infer the parameters of a predictive model; this model could be used as a component in an MPO. In grünifai example mentioned above, human feedback is used to create an MPO component of chemical desirability score.

Our first contribution is to model the chemist’s goal via probabilistic user-modeling, to automatically adapt the scoring function to match their goal. The adaptation is done by querying a chemist for feedback on molecules and using the feedback to estimate the parameters of the desirability functions of each molecular property to be optimized in Task 1, or in Task 2 for fitting a non-parametric predictive model for single-parameter optimization. We show empirically that a scoring function adapted in this way will yield molecules that better match the chemist’s goal. Our second contribution is to present how Bayesian optimization, a well-established machine learning method, efficiently chooses which molecules are shown to the chemist for the interest of adapting the objectives better and generating high-scoring molecules. From the methodological point of view, this work provides a proof of concept for interactive reward elicitation in drug design—that is, how to actively learn about the reward function of reinforcement learning by interacting with a human. We first show the effectiveness of the methods in simulated example cases, and then demonstrate the performance with a human chemist’s feedback using a graphical user interface for interaction with the system.

## Methods

**Fig. 1:**
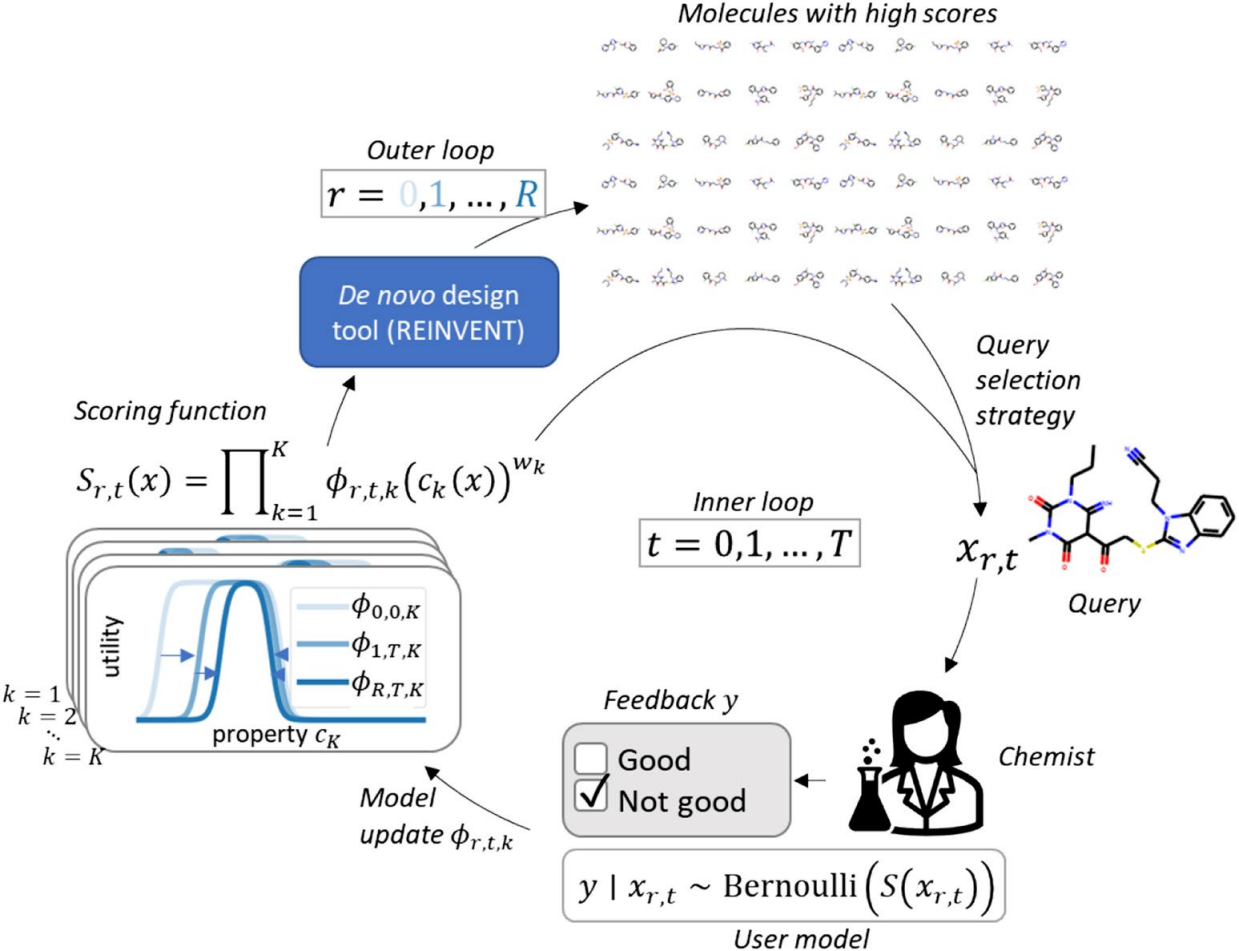
Human-in-the-loop de novo molecular design: an AI-assistant helps a chemist to decide parameters of an MPO objective function ${S}_{r,t}\left(x\right)$ iteratively at round $r$ and iteration $t$, where $r$ are rounds of goal-directed molecule generation with a de novo design tool, and $t$ are online interactions with a chemist. The objective consists of K molecular properties ${c}_{k}\left(x\right)$ with relative weights ${w}_{k}$. The utility of the $k$:th property is measured using a desirability function ${\phi }_{r,t,k}$ that defines the range of good property values. At each iteration, the method selects a molecule ${x}_{r,t}$ to query, which the chemist evaluates with feedback $y$. The method then adapts ${S}_{r,t}\left(x\right)$ based on the feedback by estimating the parameters of ${\phi }_{r,t,k}$ to match the chemist’s underlying goal

This work divides the problem of interactive adaptation of the MPO objective function into two tasks that are implemented independently: In Task 1, depicted in Fig. 1, the high-level goal is to infer the parameters of the desirability function of each property in a MPO function: A chemist inputs a set of molecular properties they wish to optimize, and their weights. What is unknown is which values of the properties are good, i.e. the desirability functions. An initial guess about the good interval of each property is given by the chemist, but they are refined by the algorithm based on the chemist’s feedback. In Task 2, the goal is to build a chemist-specific scoring component for a molecular property for single parameter optimization; the same component can later act as part of the objective in an MPO. The chemist evaluates the score of molecules with respect to the property to be optimized. This feedback is used to learn a non-parametric model, which can be used during molecular optimization to generalize the chemist's feedback to new molecules.

The proposed method for Task 1 is outlined in Fig. 1: The goal of a chemist is encoded as a composite scoring function ${S}_{r,t}\left(x\right)$ for an MPO at round $r$ of generating novel molecular designs and the $t$:th iteration of online interaction with the chemist. The scoring function consists of $K$ molecular properties ${c}_{k}\left(x\right)$ and score transformation functions ${\phi }_{r,t,k}$ that define desirability of the $k$:th molecular property. A de novo molecular design system interacts with a chemist by selecting molecules to query, and the chemist then gives feedback of how well the molecules match their goal. The feedback is used to adapt scoring function ${S}_{r,t}\left(x\right)$ so that it predicts molecules’ score more accurately, which is achieved by fitting the desirability functions ${\phi }_{r,t,k}$.

### De novo* design tool without a human-in-the-loop component*

As a *de novo* design tool we use REINVENT, which is an open-source program [4]. REINVENT uses a deep generative model to generate small molecules in the SMILES [33] format. The generative model is used in a reinforcement learning scenario, where the main objective is to maximize the score of a composite scoring function. REINVENT generates molecules by sequentially adding tokens representing atoms and their connection to a SMILES string using the generative model, also referred to as 'agent' later in the text. In reinforcement learning mode, a batch of generated SMILES strings at each epoch are scored using the scoring function. The score is used as a reward to tune the weights of the agent and thus train it to produce more high-scoring molecules.

In the current system, without HITL, the user interacts with the tool by defining the learning objective by specifying the scoring function. The scoring function of REINVENT allows the user to combine the various objectives which can play a role in molecular design. The objectives include components such as predictive models, calculated properties, 2D and 3D similarity [34, 35] and molecular docking [36]. These components are normally combined either as a weighted product

1 $$S\left( x \right) = \left[ {\mathop \prod \limits_{k = 1}^{K} \phi_{k} \left( {c_{k} \left( x \right)} \right)^{{w_{k} }} } \right]^{{{\raise0.7ex\hbox{$1$} \!\mathord{\left/ {\vphantom {1 {\mathop \sum \nolimits_{k = 1}^{K} w_{k} }}}\right.\kern-\nulldelimiterspace} \!\lower0.7ex\hbox{${\mathop \sum \nolimits_{k = 1}^{K} w_{k} }$}}}}$$

or a weighted sum

2 $$S\left(x\right)= \frac{{\sum }_{k=1}^{K}{w}_{k} {\phi }_{k}\left({c}_{k}\left(x\right)\right)}{{\sum }_{k=1}^{K}{w}_{k}}$$

where the user-selected components are denoted as ${c}_{k}$ in both equations and the corresponding weights are denoted as $w$, and *K* is the number of components. If the component outputs a continuous value, e.g. a regression model, the prediction outcome is scaled to [0, 1] using a score transformation ${\phi }_{k}$ that is the desirability function of the $k$:th property. Weights can vary in the range [1, + ∞) while the score from each component ${\phi }_{k}\left({c}_{k}\left(x\right)\right)$ can vary in the range [0, 1], resulting in an overall score within a range of [0, 1]. Both the components and the score transformations of these components are defined by the user and are manually tuned to guide the idea generation in a direction the designer assumes to be relevant to the project’s objectives.

### *Human-in-the-loop assisted *de novo* molecular design*

This section introduces two HITL methods for setting objectives in *de novo* molecular design. The first is applicable when relevant sub-objective properties are known and available as scoring components: it adapts the MPO function (Section “Adapting the MPO function using human-in-the-loop feedback (Task 1)”). The second is for cases where a specification of a scoring component for a molecular property does not exist, and we propose a method to learn a new predictive model that captures the medicinal chemist’s knowledge about the molecular property (Section "Building a new scoring component from human knowledge (Task 2)"). In both cases, the AI-assistant needs to solve an active learning problem of how to select the molecules to show to the chemist during the interaction. Different active query selection strategies are described in Section "Query selection strategies".

### Adapting the MPO function using human-in-the-loop feedback (Task 1)

This method adapts the MPO objective to match the chemist’s goal by estimating its parameters from iterative simple feedback, in the setup depicted in Fig. 1. We assume that a chemist inspects a molecule $x\in \mathcal{M}$, where $\mathcal{M}$ is the set of all valid molecules, evaluates it based on their tacit inner scoring function modelled with $S\left(x\right)$, $S:\mathcal{M}\to \left[\mathrm{0,1}\right]$, and gives binary feedback $y\in \left\{\mathrm{0,1}\right\}$. Here $y$=1 means that the molecule is good for their purpose and $y$=0 that it is not. In addition, we make the simplifying assumptions that $S$ is stationary and deterministic.

The adaptive MPO scoring function consists of $K$ adaptive scoring components ${\phi }_{r,t,k}\left({c}_{k}\left(x\right)\right)\in \left[\mathrm{0,1}\right]$, $k=1,\dots ,K$, each measuring the utility of a molecular property ${c}_{k}\left(x\right)\in {\mathbb{R}}$ that can be computed from a molecule $x$. The MPO function is adapted by modifying the desirability functions ${\phi }_{r,t,k}$, also called score transformations, at rounds of molecule generation ($r=1,\dots ,R$) and at iterations of on-line interaction with a chemist ($t=1,\dots ,T$). Let ${\theta }_{r,t,k}\in {\mathbb{R}}^{{d}_{k}}$ denote the unknown parameters of ${\phi }_{r,t,k}$, and simplify notation by writing $\phi_{k} \left( {c_{k} \left( x \right),\theta_{r,t,k} } \right): = \,\,\phi_{r,t,k} \left( {c_{k} \left( x \right)} \right)$ . The number of parameters ${d}_{k}$ depends on the model family of the transformation ${\phi }_{k}$, which is assumed to be known. In this work, we use a double sigmoid score transformation for each component, which defines a range where the generated molecules’ properties are desired to lay, with smooth thresholds. The double sigmoid transformation, illustrated in Fig. 1, is parameterized with four parameters: ${\varvec{\theta}}=\left[LOW,HIGH, {\alpha }_{1}, {\alpha }_{2}\right]$:

$$\phi \left(x,{\varvec{\theta}}\right)=\frac{{10}^{{\alpha }_{1}x}}{{10}^{{\alpha }_{1}x}+{10}^{{\alpha }_{1}LOW}}-\frac{{10}^{{\alpha }_{2}x}}{{10}^{{\alpha }_{2}x}+{10}^{{\alpha }_{2}HIGH}}$$

where $\left[LOW, HIGH\right]$ defines the desired interval of the property value $x$, ${\alpha }_{1}$ and ${\alpha }_{2}$ control the steepness of the rising and descending edge respectively.

The scores of the scoring components are aggregated using an aggregation method from Eq. (1) or (2), assuming known weights ${w}_{k}$, with a constraint that ${\sum }_{k=1}^{K}{w}_{k}=1$. The resulting adaptive scoring function ${S}_{r,t}\left(x\right)$ with Eq. (1) aggregation is

3 $$S_{r,t} \left( x \right): = \,\,S_{{{{\user2{\theta } }}_{r,t} }} \left( x \right) = \mathop \prod \limits_{k = 1}^{K} \phi_{k} \left( {c_{k} \left( x \right), \user2{\theta }_{r,t,k} } \right)^{{w_{k} }}$$

where ${{\user2{\theta } }_{r,t}=\left[{\user2{\theta } }_{r,t,1},\dots ,{\user2{\theta } }_{r,t,K}\right]}^{\mathrm{\top }}$ (bolded letter denotes a vector).

#### Task

Given $K$ molecular properties, known score transformation function family parameterized with $\user2{\theta }$, score aggregation type (Eq. (1) or (2)), score aggregation weights $w$ and an initial guess ${\user2{\user2{\theta } } }_{0}$, learn $\user2\theta$ by showing molecules to a chemist, recording their response and computing the posterior distribution of $\user2\theta$ to adapt the MPO scoring function ${S}_{{{\user2{\theta }}}}\left(x\right).$

#### Workflow

In the first round $\left(r=1\right)$, an initial batch of molecules is generated using scoring function ${S}_{{{\varvec{\theta}}}_{0}}$ as a scoring function in REINVENT. Then an active query selection strategy sequentially selects molecules to be shown to a chemist, who gives feedback to them. This continues for $T$ iterations, after which the next round begins ($r=2$) and a new batch of molecules is generated using ${S}_{{{\varvec{\theta}}}_{r-1}}$ as a scoring function, where the ${\user2{\theta } }_{r-1}={\user2{\theta } }_{r-1,T}$ is a vector of point estimates of the score transformation parameters from the last round.

#### Probabilistic model of the chemist’s score

The chemist’s unknown score is modelled using the Eq. (3), where the relevant components ${c}_{k}$ are known. We further assume that the chemist has (tacit) limits for desired values of the properties, therefore, there are two unknown parameters for each component $\user2{\theta }_{{r,t,k}} = \left[ {HIGH_{{r,t,k}} ,~LOW_{{r,t,k}} } \right]$. The two steepness parameters of the double sigmoid are assumed to be fixed.

We assume the chemist gives feedback $y$=1 with the probability $S\left(x\right)$; therefore, the observation model for the chemist’s response, given that they were shown a molecule query ${x}_{r,t}$, is

4 $$y|{x}_{r,t} \sim \mathrm{ Bernoulli}({S}_{r,t-1}({x}_{r,t}))$$

With Bayesian inference, we can then compute the posterior distribution of model parameters, conditioned on the observed data ${D}_{r,t}={\left\{\left({x}_{i},{y}_{i} \right)\right\}}_{i=1}^{{N}_{r,t}}$, where ${N}_{r,t}$ is the number of queries up to round $r$ and iteration$t$, as

5 $$p\left( {\user2{\theta } \mid D_{r,t} } \right) = \frac{{p\left( {D_{r,t} \mid \user2{\theta } } \right)p\left( \user2{\theta } \right)}}{{\smallint p\left( {D_{r,t} \mid \user2{\theta } } \right)p\left( \user2{\theta } \right)d\user2{\theta } }}$$

where $p\left( {D_{r,t} \mid \user2\theta } \right)$ is the likelihood of observed data given parameters $\user2\theta$, $p\left(\user2\theta \right)$ is the prior distribution of $\user2\theta ,$ and the denominator $\int p\left({D}_{r,t}\mid \user2\theta \right)d\user2\theta$ which normalizes the distribution is called evidence. Given the observation model in Eq. (4) and assuming that the observations are independent and identically distributed (i.i.d.), the likelihood is

6 $$p\left( {D_{{r,t}} \mid \user2{\theta }} \right) = \prod\nolimits_{{i = 1}}^{{N_{{r,t}} }} {S_{\user2{\theta }} \left( {x_{i} } \right)^{{y_{i} }} \left( {1 - S_{\user2{\theta }} \left( {x_{i} } \right)} \right)^{{1 - y_{i} }} }$$

In case an active learning query strategy selects which observations to acquire, the observations are no longer i.i.d., which in a full treatment can be taken into account. Here we make a simplifying assumption and use (6), which may result in a bias in the model.

For specifying the prior distributions $p\left(\user2\theta \right)$, the chemist provides initial values ${\user2\theta }_{0}={\left\{\left(HIG{H}_{0,k},LO{W}_{0,k}\right)\right\}}_{k=1}^{K}$ which are set to be the expected values of the prior distributions:

$$LO{W}_{k}\sim \mathrm{Normal}\left(LO{W}_{0,k},{\sigma }_{\theta ,k}^{2}\right\},$$

7 $$HIG{H}_{k}\sim \mathrm{Normal}\left(HIG{H}_{0,k}, {\sigma }_{\theta ,k}^{2}\right)$$

where ${\sigma }_{\theta ,k}=\frac{1}{8}\left(HIG{H}_{0,k}-LO{W}_{0,k}\right)$ is a hyperparameter that defines how likely the values are to differ from the initial guess, and it depends on the width of the prior belief about the desired range of the property.

Using the scoring function in REINVENT requires point estimates ${\user2\theta }_{r}$. We use the expectation of posterior distributions $\user2\theta_{{\text{r}}} = \int \user2\theta p(\user2\theta \mid {\text{D}}_{{\text{r,T}}} )d\user2\theta$, which minimizes the mean squared error of $\user2\theta$. The full algorithm is shown in Algorithm 1.
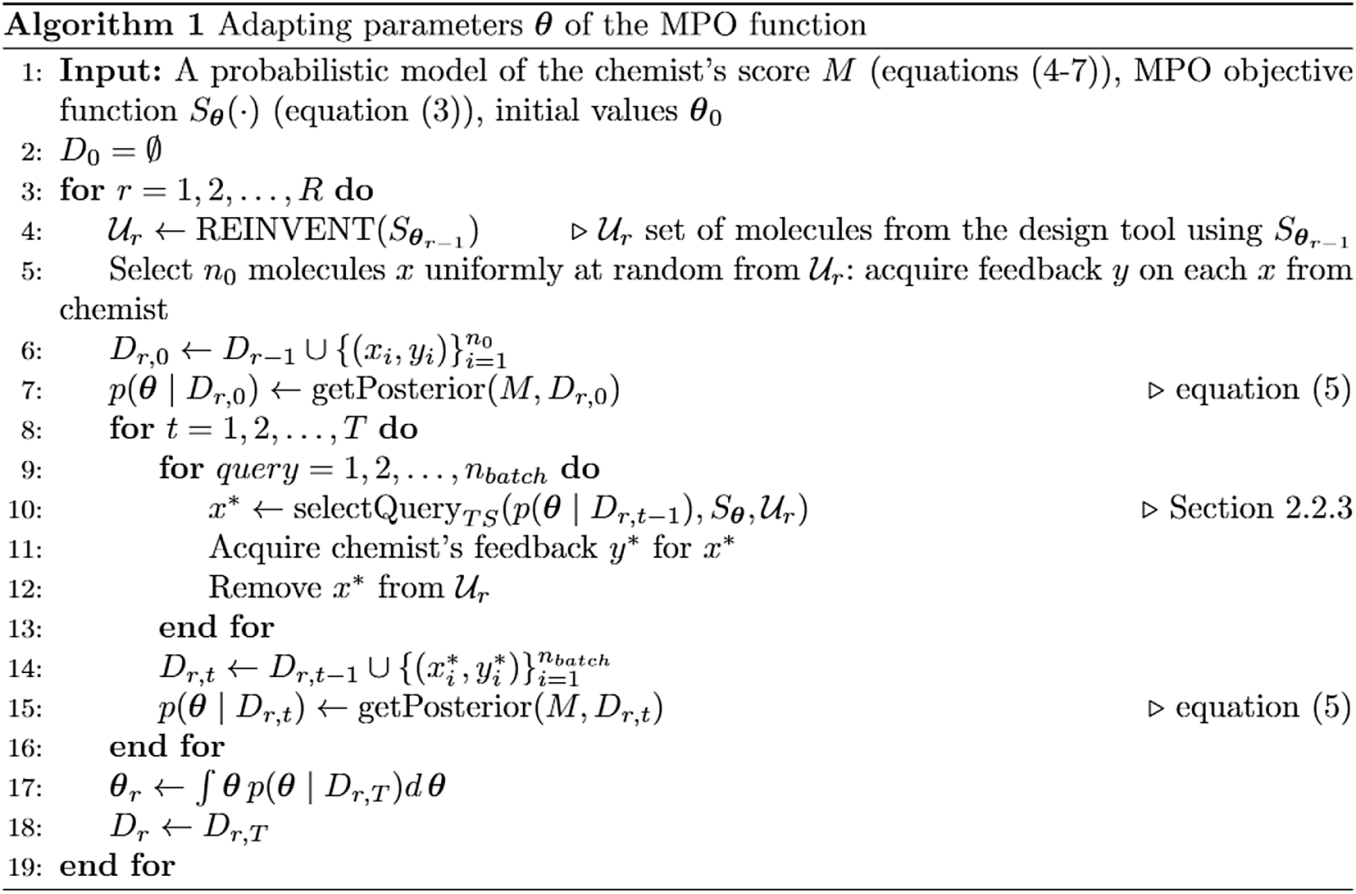


### Implementation

We use the probabilistic programming language Stan [37] to fit the model, and to compute posterior distributions and expectations of $\user2\theta$. For computational reasons, we parametrize the model with $\left(LOW, DELTA\right)$, $DELTA>0$, so that $HIGH=LOW+DELTA$. The code is publicly available at https://github.com/MolecularAI/reinvent-hitl.

#### Building a new scoring component from human knowledge (Task 2)

The second method we propose is applicable in cases where a pre-specified scoring component for a specific property is not available but, instead, the values for the molecular property of interest can be obtained via interaction with a chemist and in addition potentially in a small experimental dataset. The method learns a new predictive model from the chemist’s feedback based on the property values, and the resulting component can then subsequently be used as one of the objectives in MPO.

#### Setup

We assume a small initial dataset ${D}_{0}$ with molecules $x$ and their scores $y$ for the property of interest, either acquired beforehand from a chemist, or from experiments. In addition, there exists a pool of unlabeled molecules $\mathcal{U}$, which can be shown to a chemist. The chemist’s feedback $y$ is a score between [0,1] about the suitability of the molecule for the drug design task, with respect to the property of interest (0 = not good, 1 = very likely good). We assume a Gaussian likelihood of feedback: $y\sim N\left({f}^{*}\left(x\right),{\sigma }_{0}^{2}\right)$, where ${f}^{*}\left(x\right)$ is the chemist’s evaluation of the property of interest, and ${\sigma }_{0}$ is the standard deviation of the noise in the chemist’s answers. This means that the chemist’s answers may be erroneous but are correct on average. For simplicity, we assume that the noise in the data generating process of $y$ in ${D}_{0}$ is the same as in the chemist’s feedback. Molecules are represented by features, which in this work are descriptors such as physicochemical properties; $x\in {\mathbb{R}}^{p}$, or Morgan fingerprints $x\in {\left\{\mathrm{0,1}\right\}}^{d}$ [38], where $d$ is the dimensionality of the features.

#### Task

Given initial dataset ${D}_{0}$, a pool of unlabeled molecules $\mathcal{U}$, and a possibility to query $T$ molecules from a chemist, learn a non-parametric model $f(x$) (“a chemist’s component”) to represent the chemist’s knowledge, so that the molecules generated using $f\left(x\right)$ as a scoring function get a high chemist’s score ${f}^{*}\left(x\right)$.

#### Chemist’s component

In contrast to the previous Task 1, here in Task 2 we do not make any assumptions about the structure of the model of the chemist; instead, we use a Bayesian non-parametric model, Gaussian Process, to fit a flexible user model to ${D}_{0}$ and the feedback.

We place a Gaussian process prior on the chemist’s component, $f \sim GP\left(0,k\left(x,x^{\prime}\right)\right)$, where $k\left(x,{x}^{\prime}\right)$ is a kernel that measures the similarity of two molecules $x$ and $x^{\prime}$. The observations in data ${D}_{t}={\left\{\left({x}_{i},{y}_{i}\right)\right\}}_{i=1}^{{N}_{t}}$ include both ${D}_{0}$ and all feedback received up to iteration $t$ ($t=1,\dots ,T$), so that ${N}_{t}$ is the sum of ${N}_{0}$ and the number of feedback queries so far. The posterior of the Gaussian process, at a test point ${x}_{*}$, is then characterized with the mean ${\overline{f} }_{*}$ and variance

8 $$\bar{f}_{*} = \user2{k}_{*}^{T} \left( {\user2{K}_{t} + \sigma _{0}^{2} I} \right)^{{ - 1}} \user2{y}$$

9 $$Var\left( {f_{*} } \right) = k\left( {x_{*} ,x_{*} } \right) - ~k_{*}^{T} \left( {\user2{K}_{t} + \sigma _{0}^{2} I} \right)^{{ - 1}} \user2{k}_{*}$$

where $\user2{k}_{*}$ is a vector with elements $k\left({x}_{*},{x}_{i}\right)$, $i=1,\dots ,{N}_{t}$, and ${K}_{t}$ is a covariance matrix with entries $k\left(x,x^{\prime}\right)$ for each $x,x^{\prime}\in {D}_{t}$. The vector $y$ contains all observations ${y}_{i}$, $i=1,\dots ,{N}_{t}$. [39]

We apply two types of kernels: squared exponential for a case when $x$ are physicochemical properties, and Tanimoto kernel [40] for Morgan fingerprint features. In our experiments, Morgan fingerprints resulted in better performance, and therefore, we focus on results with them. For completeness, the results with physicochemical properties are shown in the Additional file 1: Sect. 3.2.

#### Implementation

We use GPflow [41] to implement the chemist’s component using a standard Gaussian process regression model, and Tanimoto kernel implementation from [42].

#### Query selection strategies

Active learning can be used to select a molecule for a chemist to label, from the pool of unlabeled molecules $\mathcal{U}$. In typical active learning settings, $\mathcal{U}$ is available before training. In our work, $\mathcal{U}$ either consists of molecules from a previous molecule generation, or molecules from public databases. The goal in active learning is to learn an accurate model that maps from molecules $x$ to labels $y$.

Our setup differs from standard active learning in that the model will subsequently be used as a scoring function for molecule generation (technically: a reward function in reinforcement learning), and, therefore, it is desired to have a model that can correctly identify high-scoring molecules. This leads to an exploration–exploitation trade-off in query selection: the system needs to trade off showing as many positive examples as possible, while ensuring that unknown areas are explored sufficiently to find new positive examples. We use a Bayesian optimization approach based on Thompson sampling [29] to solve this trade-off. Below we give brief summaries of the query selection strategies that we compare in this work: random sampling, uncertainty sampling, pure exploitation, and Thompson sampling. Each of them aims at selecting a next molecule ${x}^{*}\in \mathcal{U}$ to query from a chemist.

#### Random sampling

Sample ${x}^{*}$ uniformly randomly from $\mathcal{U}$.

#### Uncertainty sampling

Select the molecule that the model is the most uncertain about: $x^{*} = {\arg \max}_{x \in u} H_{\user2\theta } \left( {y \mid x} \right)$ where ${H}_{\user2\theta}$ denotes entropy when the model parameters are $\user2\theta$, and $y\mid x$ is the predicted score of molecule $x$ in the model ($y\in \left\{\mathrm{0,1}\right\}$ in Task 1 and $y\in \left[\mathrm{0,1}\right]$ in Task 2).

#### Pure exploitation

Select the molecule that maximizes the expected expert score: $x^{*} = \arg \max_{x \in U} \int {S_{\user2\theta } (x)p(\user2\theta \mid D_{r,t} )d\user2\theta }$ (Task 1), and ${x}^{*} = \arg \max_{x \in \mathcal{U}}f\left(x\right)$ (Task 2).

#### Thompson sampling

Select a molecule that greedily maximizes the expected score given a randomly drawn belief. In Task 1, this means drawing a sample ${\user2{\theta}}_{s}$ from the current posterior distribution $p\left( {\user2{\theta} \mid D_{r,t} } \right)$, and then maximizing ${x}^{*}= \arg \max_{x \in \mathcal{U}}{S}_{{{{\user2\theta}}}_{s}}\left(x\right)$ (Algorithm 2). In Task 2, we sample one realization ${{\varvec{f}}}_{s}$ of the GP posterior at points $x\in \mathcal{U}$, and select the $x$ with the largest value of the sampled function mean ${\overline{{\varvec{f}}} }_{s}\left(x\right)$ (Algorithm in Additional file 1: Sect. 1.1).
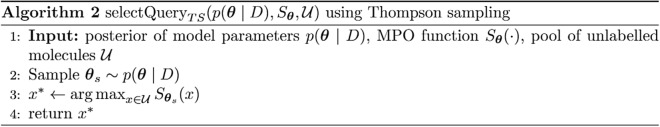


### Human-in-the-loop experiments

We demonstrate the methods in two example tasks, with binary and continuous feedback. The goal in Task 1 is to adapt the scoring function consisting of physicochemical properties to generate molecules that score high in Quantitative Estimate of Drug-likeness (QED) [10], with binary feedback. In Task 2 we train a novel scoring component for capturing the chemist’s knowledge about DRD2 activity of the molecule, based on continuous-valued feedback.

To make the study reproducible, we use an oracle to simulate the responses of a chemist instead of including a real human in the loop. Nevertheless, we assume the budget of 200 active learning queries, which is close to the maximum feasible number of interactions with a human chemist.

For evaluating how well the generated molecules match the simulated chemist’s goal, we use the score from the oracle, coined ‘oracle score’ to distinguish it from the from the score of a molecule in a scoring function. To reduce computation time in the experiments, we select queries sequentially in batches, by greedily selecting the ${n}_{\text{batch}}$ best molecules according to a query strategy at iteration $t$. As a result, the performance of other methods may be underestimated compared to random sampling, because they will not be able to optimize their selection during a batch.

#### Task 1: Adapting the parameters of the MPO function

We experimentally evaluate the method for adapting MPO in a task of generating molecules with a high QED-score [10], based on scoring components of physicochemical properties. We chose QED-score as the goal because it is inspired by how humans evaluate the drug-likeness of molecules. This makes it a suitable proxy to simulate a chemist’s intuition and, furthermore, there exists a publicly available method for approximating it [10]. We make a minor modification to the standard QED score, so that the modified score ${S}_{\text{mQED}}\left(x\right)\in \left[\mathrm{0,1}\right]$ favors smaller values of partition coefficient (logP), to make the task more difficult a priori (for the details of the modification of the desired value of logP from the original average 3 to average 1.5, see Additional file 1: Sect. 2.1).

The scoring components include the following seven physicochemical properties, calculated with RDKit [43]: molecular weight (MW), partition coefficient (SlogP), hydrogen bond donors (Lipinski) (HBD), hydrogen bond acceptors (Lipinski) (HBA), polar surface area (PSA), number of rotatable bonds and the number of aromatic rings. We assume that all properties are transformed with the double sigmoid function, with unknown HIGH and LOW parameters. The other two parameters of the double sigmoids are set to fixed values deemed good for each property based on prior knowledge, provided in Additional file 1: Section 2.2. We aggregate the scores using weighted geometric average (eq. (1)).

As a starting point in the first round, we use poor guesses on the parameters ${{\varvec{\theta}}}_{0}={\left\{\left(HIG{H}_{0,k},LO{W}_{0,k}\right)\right\}}_{k=1}^{7}$ to create a scoring function that gives high score to molecules with a wide range of molecular properties. The exact initial values are reported in Additional file 1: section 2.3 We use this scoring function in REINVENT and collect the high-scoring molecules generated during 300 epochs of training as the first unlabeled molecules $\mathcal{U}$ (depending on the run, this results in the order of 1,000–10,000 molecules). The number of epochs was chosen so that in most cases a (local) maximum has been found, observed as flattening of the learning curve. We run the experiment for two rounds (initialization, and two rounds of feedback queries, $R=2$), and evaluate the performance as the average oracle score of the generated molecules at initialization and at the end of each round.

At each round, the user model is initialized with $10$ randomly chosen molecules, and the priors of the user-model are defined by the previous round’s ${\theta }_{r-1}$ (${\theta }_{0}$ in the first round). Then, we do $10$ iterations of querying ${n}_\text{batch}=10$ molecules in batches. This means that we query in total $T=110$ molecules from a simulated chemist, making the total query budget in the experiment 220. The simulated chemist gives feedback 1 randomly with probability of ${S}_\text{mQED}\left(x\right)$. For each query strategy described in "Task 2: Learn human knowledge about a molecular property as a separate component" Section we repeat the experiment ten times with different random seeds to quantify variance due to different ${D}_{0}$ and stochasticity in the simulated chemist’s answers.

#### Task 2: Learn human knowledge about a molecular property as a separate component

We test possibility to learn human knowledge using example of DRD2 activity. For reproducibility, we use an oracle model, instead of a human chemist. We derive human component *f(x)* using algorithm described in "Building a new scoring component from human knowledge (Task 2)" Section. To evaluate derived human component *f(x)*, we first use *f(x)* as a scoring function in REINVENT to train an agent; we then sample molecules from trained REINVENT agent, and evaluate sampled molecules using the oracle model.

To compare query strategies, we derive human component *f(x)* for each of the query strategies described in "Building a new scoring component from human knowledge (Task 2)" Section, and repeat the experiments 10 times with different random seeds.

For sensitivity analysis, we repeat the experiment, but this time we derive human component *f(x)* with noise added to the simulated chemist’s answers.

##### Training oracle model

We evaluate the Task 2 method in an example case of learning the DRD2 activity of molecules from feedback. For an oracle in this case, we use activity prediction model trained on a large publicly available dataset on DRD2 activity [44]. We used an activity prediction model trained on both the active and inactive compounds of the ExcapeDB DRD2 modulator set.^1^ To train the model, stereochemistry was stripped from all compounds in the dataset, and they were represented in their canonical form by using RDKit [43]; the resulting duplicates were removed; data was split to test and training sets with a stratified split and the compounds were represented with ECFP6 fingerprint (radius 3) hashed to 2048 bits; a Scikit-learn [45] Random Forest Classifier model was trained to discriminate active from inactive compounds; Optuna [46] was used for finding the optimal hyperparameters with a 5-fold cross validation; the performance of the resulting model in terms of area under the curve (AUC) was 0.945. We use predicted positive class probabilities from the activity prediction model when answering the queries.

##### Deriving human component f(x)

As described in "Task 1: Adapting the parameters of the MPO function" Section, we derive human component as a Gaussian Process model. The DRD2 dataset consists of 275,768 molecules represented as SMILES strings. In the beginning, we sample randomly ${N}_{0}=10$ molecules to be the initial dataset ${D}_{0}$ and acquire their scores from the simulated chemist (oracle in the noise-free case). The rest of the molecules are used as unlabeled molecules $\mathcal{U}$ in the simulated experiments. During interaction, we query in total 200 molecules from the simulated chemist in batches of 5 ($T=40$).

##### Sensitivity analysis

In addition to a noise-free case, we do a sensitivity analysis of the method with respect to the noise in the simulated chemist’s answers. For this, the oracle’s answers are corrupted with independent Gaussian noise with standard deviation ${\upsigma }_{0}=0.15$ (moderate noise) and ${\upsigma }_{0}=0.30$ (severe noise). For simplicity, feedback values are capped within range [0,1].

For evaluation, we set $f\left(x\right)$ as the scoring function in REINVENT and train the agent for 300 epochs. After obtaining a trained agent, we sample 1024 molecules from the agent and evaluate sampled molecules using the oracle model. For each query strategy described in "Building a new scoring component from human knowledge (Task 2)" section, we repeat the experiments 10 times with different random seeds.

#### Deriving a DRD2 scoring function using a human

**Fig. 2:**
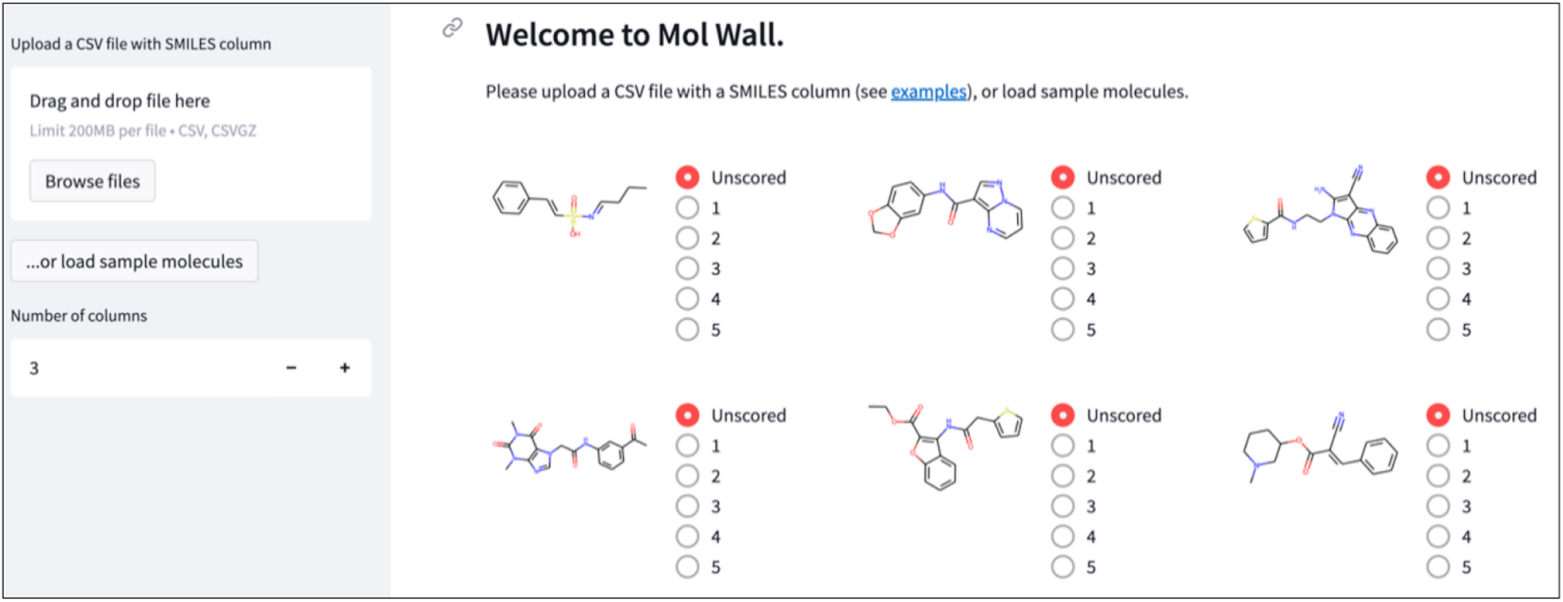
Graphical user interface for giving feedback to molecules. The chemist evaluates DRD2 activity of molecules on a scale from 1 to 5 For initialization, we randomly sample 10 molecules and get their scores from the oracle. For the experiment with a human chemist, we randomly sample 10,000 molecules to be unlabeled molecules $\mathcal{U}$ to speed up the method. For ten iterations we sequentially query 100 molecules in batches of 10 from a chemist, who evaluates them on a scale from 1 to 5 (0 = very likely not active, 5 = very likely active). The scores are linearly scaled to the range [0,1]. The order of the evaluated molecules is chosen using Thompson sampling that was the best in the simulated experiments. For evaluating the performance, the oracle model is used to score the molecules generated by REINVENT with the chemist’s component as a scoring function at iteration $t=1, \dots ,10$.

To show that the results of the simulated experiment in Task 2 are relevant, we exemplified the method with human feedback in a modified version of Task 2 where the chemist was queried directly. We let a medicinal chemist (who is also coauthor of the manuscript) interact with the system in the same DRD2 activity setup as described in "Task 2: Learn human knowledge about a molecular property as a separate component" Section. The system has a graphical user interface for interaction, shown in Fig. 2.

## Results

### Task 1: Adapting the parameters of the MPO objective function

**Fig. 3:**
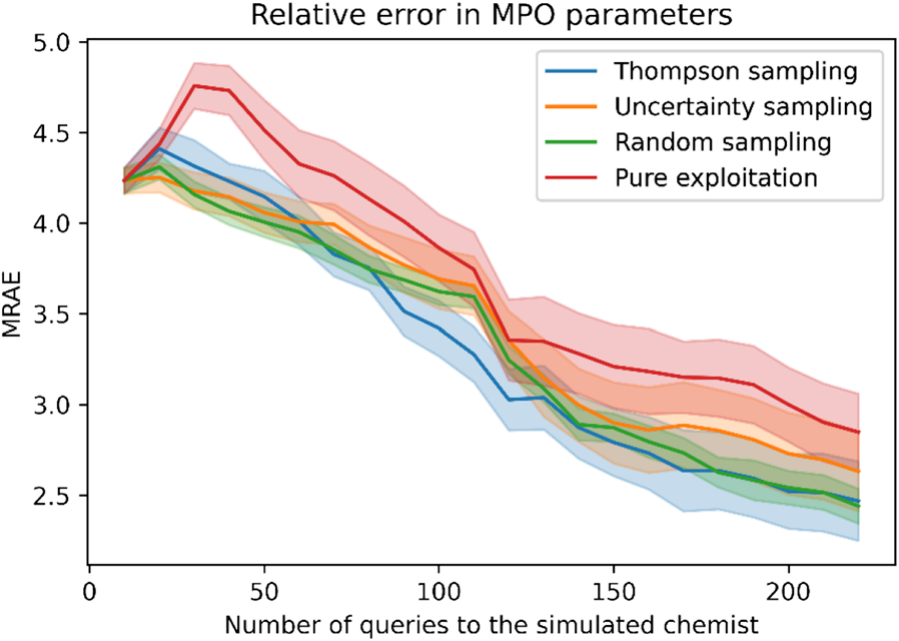
The parameters of the MPO objective are better estimated with increasing amount of feedback. The mean relative absolute error (MRAE) in the estimated parameters decreases with increasing human feedback, and fastest with Thompson sampling. Solid lines show average of MRAE over 10 random seeds, and the shaded areas one standard error of the mean (SEM)

**Fig. 4:**
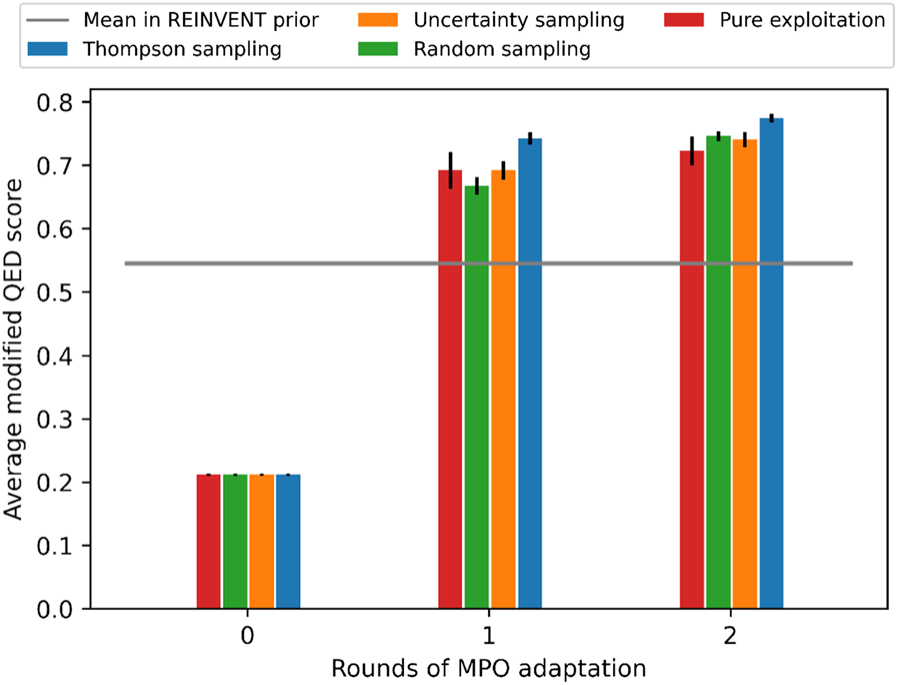
The average oracle score of the generated molecules increases at each round of adapting the MPO. At each round, a new batch of molecules is generated using an adapted scoring function after in total 110 queries (round 1) and 220 queries (round 2) to a simulated chemist. For comparison, we show round 0 that is the performance with the initial guess ${\theta }_{0}$. The bars show the mean of the average oracle score of the generated molecules over 10 random seeds, and the error bars represent one SEM. The gray horizontal line shows the average oracle score in 5000 molecules sampled from REINVENT without MPO objective, using its prior agent

A probabilistic model of the chemist’s score can estimate the unknown parameters of the desirability functions sufficiently well from the feedback, and as a result, the adapted MPO scoring function achieves improved QED score in the generated molecules after just one round and 100 HITL interaction. The uncertainty in the model decreases after feedback (Additional file 1: Section 3.1), and as a result, the error in the estimated MPO parameters decreases with increasing feedback, shown in Fig. 3. The adapted scoring function also improves the quality of the generated molecules at each round, as seen in the increase in the average oracle score in Fig. 4, which is the main objective of the method. All query selection strategies are effective in increasing the performance.

To study whether introducing HITL is making significant difference in Task 1, we compared the average QED score produced using different query strategies at round 1 to the baseline (average QED score in 1000 molecules sampled from REINVENT prior, in 10 repetitions) by analysis of variance. The average QED scores of all query strategies were significantly different from the baseline: p-value in ANOVA $$ 8.7^*10^{-9}$$, and adjusted p-values in Tukey's test <0.05 for all query strategies compared to the REINVENT prior (values reported in the Additional file 1).

To study which query strategy is better, we compared the performance of the query strategies using the area under the elicitation curve as a test statistic. We use the analysis of variance (ANOVA) and post-hoc Tukey's HSD test for pairwise comparisons, to test for statistical significance. For Task 1, we find no statistically significant difference between different query strategies (p-value in ANOVA 0.196, p-values adjusted for multiple comparisons in Tukey's test are reported in the Additional file 1). In Task 2, however, there are significant differences between the performance of query strategies, see next section.

### Task 2: New scoring component for human knowledge

**Fig. 5:**
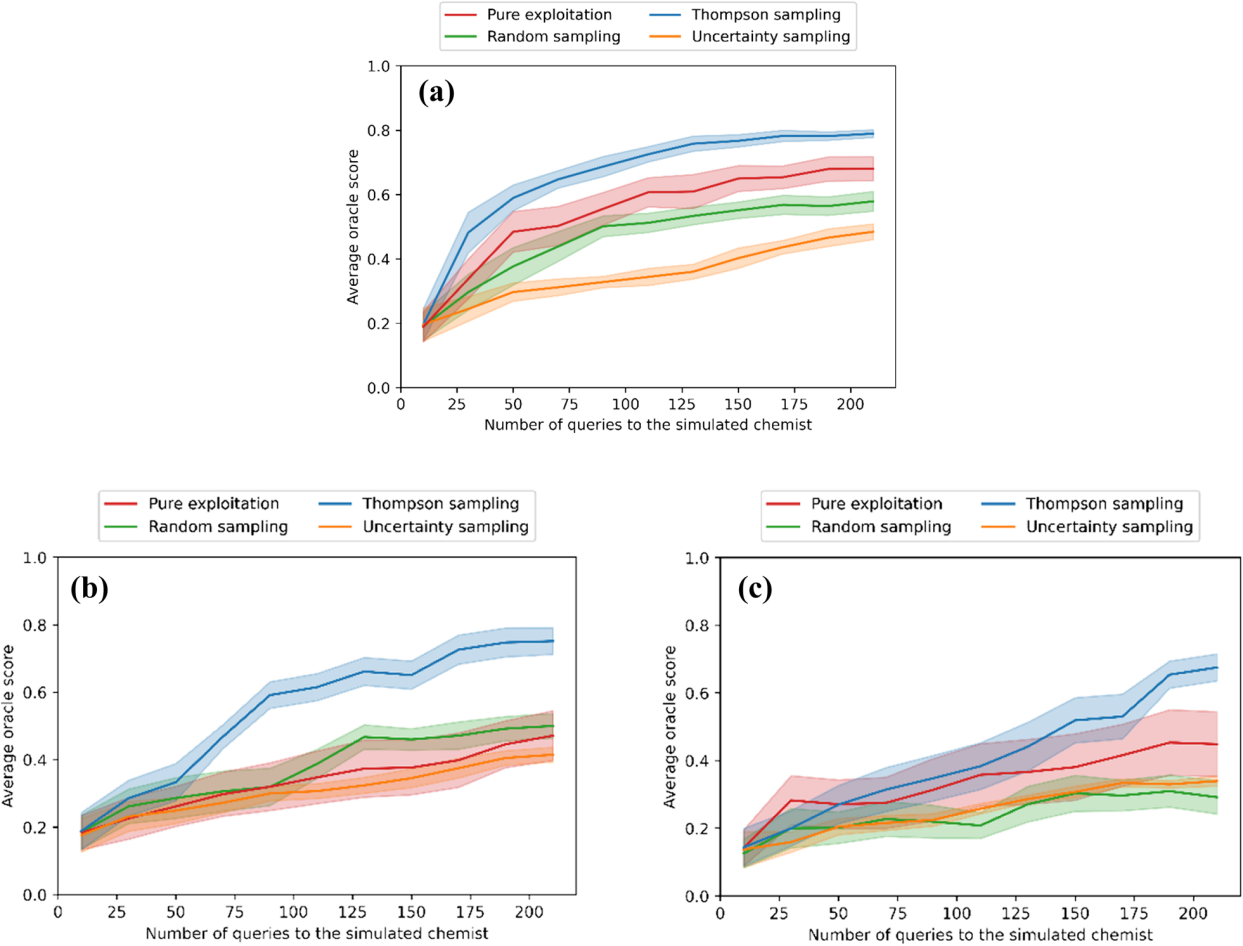
A non-parametric scoring component that represents the chemist’s knowledge improves REINVENT output even with small number of queries (< 100) to a simulated chemist. The lines show the average oracle score in the REINVENT output and shaded areas its variation in 10 repeated experiments (mean and SEM). The method is not very sensitive to Gaussian noise in the simulated chemist’s answers. **a** noise level ${\sigma }_{\mathrm{chemist}}=0.0$, **b** ${\sigma }_{\mathrm{chemist}}=0.15$, **c** ${\sigma }_{\mathrm{chemist}}=0.30$

Similar results are obtained in the Task 2. Figure 5a shows that the average oracle score of the generated molecules increases with increasing amount of feedback from a simulated chemist, with all query selection methods. Thompson sampling outperforms the other approaches and takes on average less than 70 queries to achieve the average oracle score 0.6, and less than 170 queries to achieve score close to 0.8, in the noise-free case. It should be noted that the first 10 molecules are always chosen using random sampling to initialize the model, and iteration 1 corresponds to the subsequent first interaction with the simulated chemist. In case the simulated chemist’s answers contain noise (Fig. 5 b,c), Thompson sampling reaches performance 0.6 in less than 110 (190) queries for noise level ${\sigma }_{\mathrm{chemist}}=0.15$ ($0.30$).

We use analysis of variance (ANOVA) and post-hoc Tukey's HSD test to test for statistical significance of the results. For no-noise case (${\sigma }_{\mathrm{chemist}}=0$), all query strategies were significantly different from each other except for pure exploitation to random sampling (ANOVA: p-value 7⋅10^–8^; the adjusted p-values of Tukey's test are reported in the Additional file 1). However, as noise increases in the simulated chemist's answers, the difference between methods becomes less evident: for noise level ${\sigma }_{\mathrm{chemist}}=0.15$, only Thompson sampling is significantly different from other methods (ANOVA: p-value 0.002; Adjusted p-values in Tukey's test: 0.009 for Thompson sampling vs. Pure exploitation, 0.043 for Thompson sampling vs. Random sampling and 0.002 for Thompson sampling vs. Uncertainty sampling; the rest of the p-values are reported in the Additional file 1). For ${\sigma }_{\mathrm{chemist}}=0.30$ case, none of the methods were significantly different from each other (p-value in ANOVA 0.109).

### Human interaction

**Fig. 6:**
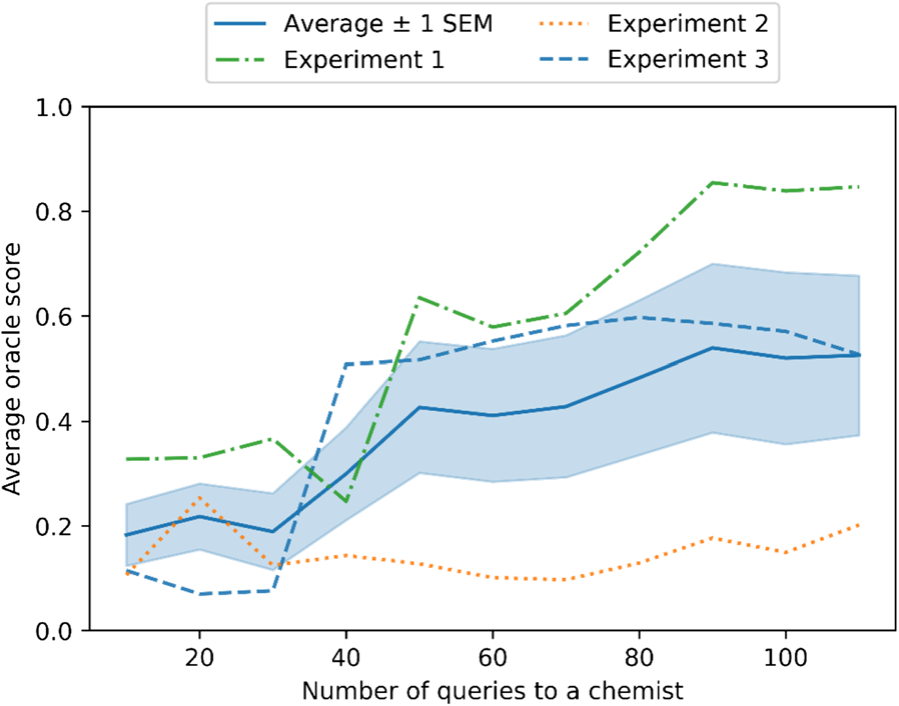
A medicinal chemist’s feedback on DRD2 activity of molecules improves the average activity of the generated molecules, measured using activity prediction model described in “Task 2: Learn human knowledge about a molecular property as a separate component” section. The dashed lines show performance in three repeated experiments. The performance is summarized in mean performance (solid line) with one standard error of mean (shaded area). The repetitions differ by different randomly sampled initial data and consequently different actively selected queries. The queries are selected using Thompson sampling

A medicinal chemist’s feedback achieves similar performance as the simulated chemist’s feedback in the previous section. On average, the performance increases from 0.18±0.06 to 0.52±0.15 (mean± standard error of the mean). In the best case in Figure 6, the average DRD2 activity evaluated by the oracle model (see "Task 2: Learn human knowledge about a molecular property as a separate component" Section) increases 159% (from 0.33 to 0.85) in the generated molecules after 100 queries to the chemist. In the two other repetitions, initial performance was lower, and in one case the method was not able to improve performance due to poor initial data. Different randomly chosen initial data caused different queries and hence also different performance. According to the chemist, the molecules shown in the first (experiment #1: green) experiment were easier to evaluate because there were more active molecules than in the ones shown in the latter two repetitions (experiment #2: orange and experiment #3: blue).

The same medicinal chemist interacted with the system in all three experiments, and therefore factors such as learning or fatigue could affect the results. We could not systematically evaluate these effects; however, the experiment #1 was the best and the experiment #3 the second best, so we did not observe any consistent effect of learning or fatigue in this demonstration.

## Discussion and conclusion

This work presents the first proof-of-principle for using human-in-the-loop interactions to aid *de novo* molecular design. We studied two approaches which we envision captures the basic use-cases where interactive machine learning provides a more principled way to exploit chemist’s knowledge than manual trial and error. The first approach tunes the parameters of reward function components, and could reduce users’ mental load of planning the scoring function that captures their goal, which might be especially useful for new users of *de novo* tools. The second approach builds a scoring function from scratch, and could benefit at the start of new projects, where experimental datasets may exist but are very small, and we wish to augment them with chemists’ intuition.

Although the second approach could be directly used to find a non-parametric MPO objective function in principle, we believe the first method to be more efficient for that task, because it allows leveraging existing domain knowledge. The domain knowledge comes from two sources: the chemist explicitly defines which molecular properties are of interest, and, more importantly, the method predicts molecular properties using pre-trained models, which compress chemical knowledge from large databases.

The experimental results quantify the improvement in *de novo* molecule generation with human-in-the-loop interaction. Experiments with a simulated chemist show that less than 200 molecule evaluations are sufficient to significantly improve the average score of the generated molecules in both use-cases (Tasks 1 and 2), even with noisy feedback. Furthermore, a demonstration with a medicinal chemist’s feedback supports this conclusion. We also provide a graphical user-interface where a chemist can interactively input their feedback.

It is known that medicinal chemist’s evaluation of molecules varies greatly between individuals and the answers are sometimes not consistent even for one person [47]. Our probabilistic model tackles this using a noise model and by assuming that, on average, the answers are correct. This assumption may be sufficient in simple cases, and necessary for cases with little data. However, the chemist’s feedback is likely to include biases, as they are ubiquitous in any human assessment. Ways to address the bias by soliciting answers from multiple experts have been studied in expert knowledge elicitation [48]. Another possibility could be to learn about the biases of the experts, by using cognitive models and estimating their parameters to model the user behavior. To our knowledge this has not yet been done in HITL tasks.

## Supplementary Information

**Additional file 1: Figure S1.** Modified desirability function of octanol-water partition coefficient. The weights of components in the modified QED score are the mean weights ${\mathrm{QED}}_{\mathrm{w}}^{\mathrm{mo}}$ from [1], except that the weight of ALERTS component is set to 0.0 to remove the dominating effect of structural alerts. **Figure S2.** Visualization of posterior distributions of the desired interval $[LOW,HIGH]$ of seven physicochemical properties (vertical panels) (a) after initialization with 10 randomly selected queries and (b) after 100 queries to an oracle. Colored vertical lines show samples from posteriors of parameters $LOW$ (blue) and $HIGH$ (red). Light blue dots represent molecules and their true scores in each desirability function. Expected value of the parameters is visualized with vertical black lines, showing that the desired interval is refined and narrowed down during interaction. Furthermore, the uncertainty about parameters decreases after interaction. **Table S1.** Values of fixed parameters of the double sigmoid desirability functions in Task 1 experiments. **Table S2.** Initial values of the parameters that are adapted during user interaction in Task 1.

## Data Availability

The algorithms, source code and datasets used to produce the results in this article are available in ReinventHITL repository https://github.com/MolecularAI/reinvent-hitl and the graphical user interface in https://github.com/MolecularAI/molwall. The DRD2-dataset supporting the conclusions of this article is available in the ReinventCommunity repository, https://github.com/MolecularAI/ReinventCommunity.

## Abbreviations

AI: Artificial intelligence;
DMTA: Design-make-test-analyze;
DRD2: Dopamine receptor D_2_;
GAN: Generative adversarial network;
GP: Gaussian process;
HBA: Hydrogen bond acceptors;
HBD: Hydrogen bond donors;
HITL: Human-in-the-loop;
MPO: Multi-parameter optimization;
MRAE: Mean relative absolute error;
MW: Molecular weight;
PSA: Polar surface area;
SEM: Standard error of the mean;
SlogP: Partition coefficient;
SMILES: Simplified molecular-input line-entry system;
QED: Quantitative estimate of drug-likeness.

## Footnotes

1 The DRD2 activity prediction model is available from https://github.com/MolecularAI/ReinventCommunity.
