# Supplementary material for: Human-in-the-loop assisted de novo molecular design
Source: J Cheminform. 2022 Dec 28;14:86. doi: 10.1186/s13321-022-00667-8 (PMC9795720; doi:10.1186/s13321-022-00667-8)
Supplement: Supplementary file 1 — Additional file 1: Figure S1. Modified desirability function of octanol-water partition coefficient. The weights of components in the modified QED score are the mean weights ${\mathrm{QED}}_{\mathrm{w}}^{\mathrm{mo}}$QEDwmo from [1], except that the weight of ALERTS component is set to 0.0 to remove the dominating effect of structural alerts. Figure S2. Visualization of posterior distributions of the desired interval $[LOW,HIGH]$[LOW,HIGH] of seven physicochemical properties (vertical panels) (a) after initialization with 10 randomly selected queries and (b) after 100 queries to an oracle. Colored vertical lines show samples from posteriors of parameters $LOW$LOW (blue) and $HIGH$HIGH (red). Light blue dots represent molecules and their true scores in each desirability function. Expected value of the parameters is visualized with vertical black lines, showing that the desired interval is refined and narrowed down during interaction. Furthermore, the uncertainty about parameters decreases after interaction. Ta [file 13321_2022_667_MOESM1_ESM.docx]

Additional file for Human-in-the-Loop Assisted *de Novo* Molecular Design

Iiris Sundin^1,^^[[1]](#footnote-1)^*, Alexey Voronov^2,*^, Haoping Xiao^1^, Kostas Papadopoulos^2,^^[[2]](#footnote-2)^†, Esben Jannik Bjerrum^2,†^, Markus Heinonen^1^, Atanas Patronov^2,†^, Samuel Kaski^1,3^, Ola Engkvist^2,4^

^1^ Department of Computer Science, Aalto University, Espoo, Finland

^2^ Molecular AI, Discovery Sciences, R&D, AstraZeneca, Gothenburg, Sweden

^3^ Department of Computer Science, University of Manchester, Manchester, United Kingdom

^4^ Department of Computer Science and Engineering, Chalmers University of Technology, Gothenburg, Sweden

# Machine learning methods

## Task 2: Chemist’s component and Thompson sampling

Algorithm for learning a chemist’s components in Task 2 is shown in Algorithm S1. It includes the human-in-the-loop interaction (lines 3-12) and Thompson sampling query selection strategy (lines 5-6).


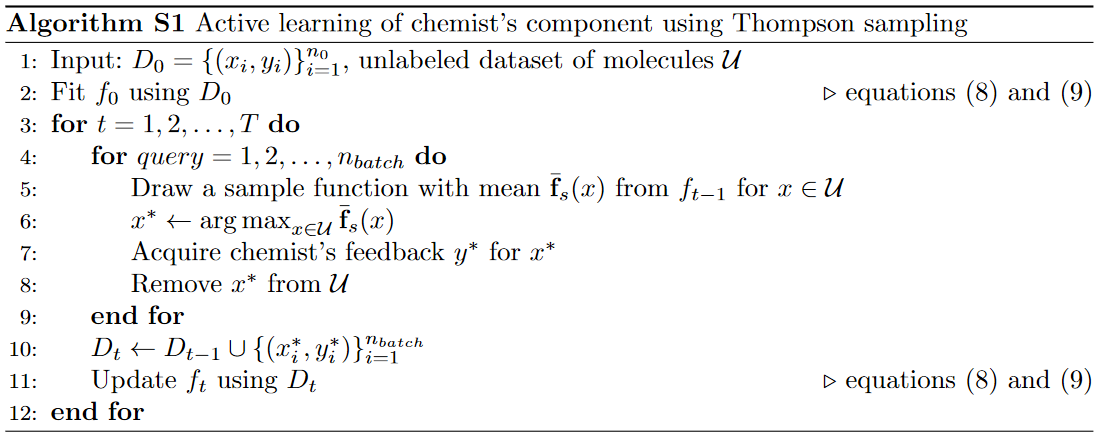


# Experiments

## Modified QED score in Task 1

In the experiments of Task 1, we set the simulated chemist’s goal to be the druglikeness of a molecule. To make the goal harder to achieve a priori, we modify the Quantitative Estimate of Druglikeness (QED) score from [1] to favor slightly lower values of octanol-water partition coefficient AlogP, using the rdkit implementation of QED score^[[3]](#footnote-3)^. Figure S1 compares the original desirability function of AlogP from [1] with the modified desirability function that has been shifted towards zero by 1.5 units.


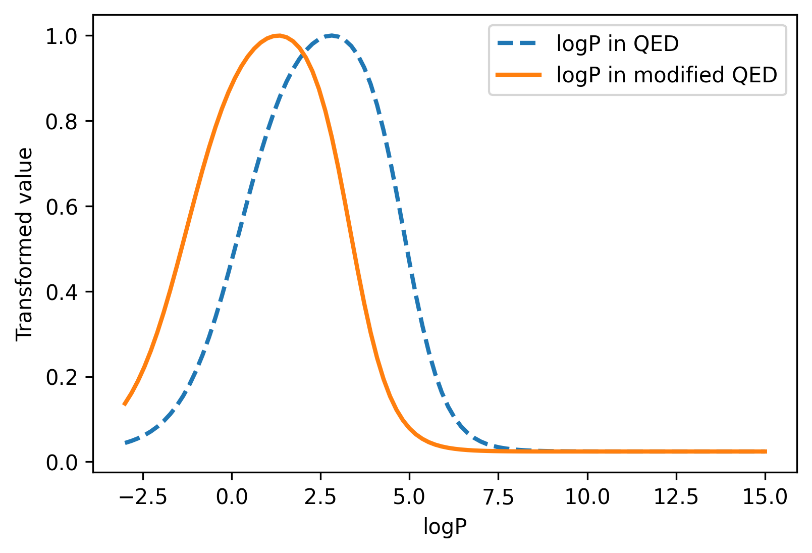


Figure S1. Modified desirability function of octanol-water partition coefficient.

The weights of components in the modified QED score are the mean weights $QED_{w}^{mo}$ from [1], except that the weight of ALERTS component is set to 0.0 to remove the dominating effect of structural alerts.

## Parameters of the scoring components in Task 1

In the Task 1, the steepness parameters of double sigmoid desirability functions are specified by domain knowledge, to avoid potential identifiability issues. The values used in experiments for each physicochemical property are listed in the Table S1, using parameterization from REINVENT scoring package^[[4]](#footnote-4)^; the parameters are transformed to $\alpha_{1}$ and $\alpha_{2}$ as $\alpha_{1}=\frac{coef\_se}{coef\_div}$ and $\alpha_{2}=\frac{coef\_si}{coef\_div}$. For simplicity, the component weights are assumed to be equal, even though this results in a mismatch between the model and the goal (modified QED score, see Section 2.1 of Supplementary material); in our early experiments we found that the weights have only minor effect on the results.

Table S1. Values of fixed parameters of the double sigmoid desirability functions in Task 1 experiments

| Molecular property | $coef\_se$ | $coef\_si$ | $coef\_div$ | Component weight $w$ |
| --- | --- | --- | --- | --- |
| Molecular weight | 2 | 2 | 175.77 | 1 |
| SlogP | 2 | 2 | 3.0 | 1 |
| HB-donors (Lipinski) | 2 | 2 | 2.41 | 1 |
| HB-acceptors (Lipinski) | 4.4 | 2 | 4.42 | 1 |
| PSA | 2 | 2 | 75.34 | 1 |
| Number of rotatable bonds | 2 | 2 | 5.69 | 1 |
| Number of aromatic rings | 2 | 2 | 2.28 | 1 |

## Initial values of parameters in Task 1

The initial parameters $\theta_{0}$ describing the desired interval $[LOW_{0}, HIGH_{0}]$ for each molecular property are given in Table S2. The values of these parameters are modified during interaction, resulting in adaptation of the MPO objective function to match with the chemist’s goal. The intervals in Table 2 are intentionally set wide, to demonstrate that the method is able to narrow down the desired range even from poor initial values.

Table S2. Initial values of the parameters that are adapted during user interaction in Task 1

| Molecular property | $LOW_{0}$ | ${HIGH}_{0}$ |
| --- | --- | --- |
| Molecular weight | 50 | 700 |
| SlogP | 3 | 10 |
| HB-donors (Lipinski) | 1 | 8 |
| HB-acceptors (Lipinski) | 2 | 11 |
| PSA | 100 | 300 |
| Number of rotatable bonds | 5 | 20 |
| Number of aromatic rings | 1 | 10 |

## Tanimoto kernel

For completeness, this section describes Tanimoto kernel introduced in [2]. The molecules $x$ are represented using Morgan fingerprints (radius 3, 1024 bits), and the kernel defines similarity between two molecules as

$$k_{Tanimoto}\left( x,x’ \right)=\sigma^{2}\frac{<x,x^{'}>}{<x,x>+<x',x'>-<x,x'>},$$

(S1)

where $\sigma^{2}$ is prior variance at $x$ and $<x,x^{'}>$ is an inner product of two vectors. In case of bit vectors such as Morgan fingerprints, it is equivalent to the number of overlapping bits between $x$ and $x'$, which means that the kernel computes the ratio between intersection and union of the fingerprints.

## Tests of statistical significance

For the test on whether there is statistically significate difference between using HITL and not using HITL in Task 1, below are adjusted p-values for comparing performance to REINVENT prior:

- Pure exploitation vs. REINVENT prior: 0.001,
- Random sampling vs. REINVENT prior: 0.001,
- Thompson sampling vs. REINVENT prior: 0.001,
- Uncertainty sampling vs. REINVENT prior: 0.001.

In this comparison, which only considers performance at round 1, the performance of Thompson sampling is significantly different from Random sampling (adjusted p-value 0.0254).

For the test on whether there is statistically significant difference between sampling strategies in Task 1, below are Adjusted p-values in post-hoc Tukey's HSD (honestly significant difference) test:

- Pure exploitation - Random sampling: 0.2068,
- Pure exploitation - Thompson sampling: 0.3171,
- Pure exploitation - Uncertainty sampling: 0.7898,
- Random sampling - Thompson sampling: 0.9,
- Random sampling - Uncertainty sampling: 0.6654,
- Thompson sampling - Uncertainty sampling: 0.8074.

For the test on whether there is statistically significant difference between sampling strategies in Task 2, below are Adjusted p-values in post-hoc Tukey's HSD (honestly significant difference) test, for different noise levels:

σ_chemist_ = 0:

- Pure exploitation - Random sampling: 0.2464,
- Pure exploitation - Thompson sampling: 0.0345,
- Pure exploitation - Uncertainty sampling: 0.001,
- Random sampling - Thompson sampling: 0.001,
- Random sampling - Uncertainty sampling: 0.0377,
- Thompson sampling - Uncertainty sampling: 0.001.

σ_chemist_ = 0.15:

- Pure exploitation - Random sampling: 0.9 (not significant),
- Pure exploitation - Thompson sampling: 0.0088 (significant),
- Pure exploitation - Uncertainty sampling: 0.9 (not significant),
- Random sampling - Thompson sampling: 0.0434 (significant),
- Random sampling - Uncertainty sampling: 0.6836 (not significant),
- Thompson sampling - Uncertainty sampling: 0.0026 (significant).

σ_chemist_ = 0.30:

- Pure exploitation - Random sampling: 0.5707,
- Pure exploitation - Thompson sampling: 0.7598,
- Pure exploitation - Uncertainty sampling: 0.6661,
- Random sampling - Thompson sampling: 0.1354,
- Random sampling - Uncertainty sampling: 0.9,
- Thompson sampling - Uncertainty sampling: 0.1879.

# Additional results

## Posterior distributions of desirability function parameters

During interaction with a chemist in Task 1, the desired range of properties is refined and uncertainty about the parameters of the adaptive scoring function is reduced. Fig S2 visualizes how uncertainty about the parameters of desirability functions decreases in the first round after feedback from an oracle. The posterior distributions of $LOW$ (blue) and $HIGH$ (red) become more concentrated, which is seen as more dense color at the edges of the desired intervals.


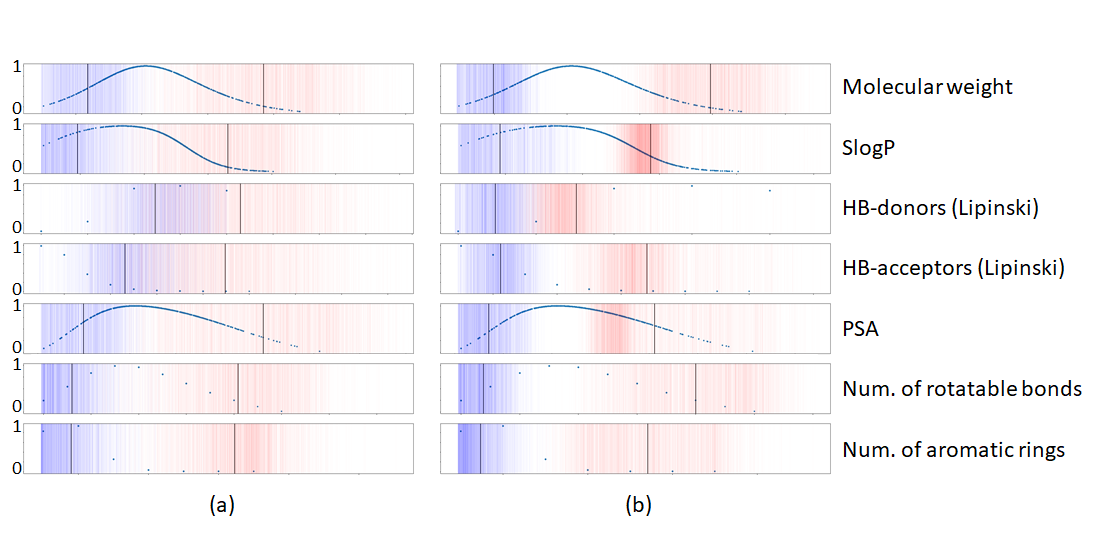


Figure S2. Visualization of posterior distributions of the desired interval $[LOW,HIGH]$ of seven physicochemical properties (vertical panels) (a) after initialization with 10 randomly selected queries and (b) after 100 queries to an oracle. Colored vertical lines show samples from posteriors of parameters $LOW$ (blue) and $HIGH$ (red). Light blue dots represent molecules and their true scores in each desirability function. Expected value of the parameters is visualized with vertical black lines, showing that the desired interval is refined and narrowed down during interaction. Furthermore, the uncertainty about parameters decreases after interaction.

# References

[1] G. R. Bickerton, G. v. Paolini, J. Besnard, S. Muresan, and A. L. Hopkins, “Quantifying the chemical beauty of drugs,” *Nat Chem*, vol. 4, no. 2, p. 90, Feb. 2012, doi: 10.1038/NCHEM.1243.

[2] L. Ralaivola, S. J. Swamidass, H. Saigo, and P. Baldi, “Graph kernels for chemical informatics,” *Neural Networks*, vol. 18, no. 8, pp. 1093–1110, Oct. 2005, doi: 10.1016/J.NEUNET.2005.07.009.

1. * Correspondance: [iiris.sundin@aalto.fi](mailto:iiris.sundin@aalto.fi), [alexey.voronov1@astrazeneca.com](mailto:alexey.voronov1@astrazeneca.com) [↑](#footnote-ref-1)
2. † Currently at Odyssey Therapeutics, Cambridge, MA, USA [↑](#footnote-ref-2)
3. https://www.rdkit.org/docs/source/rdkit.Chem.QED.html [↑](#footnote-ref-3)
4. https://github.com/MolecularAI/reinvent-scoring [↑](#footnote-ref-4)
